# NRAMP1, VDR, HLA-DRB1, and HLA-DQB1 Gene Polymorphisms in Susceptibility to Tuberculosis among the Chinese Kazakh Population: A Case-Control Study

**DOI:** 10.1155/2013/484535

**Published:** 2013-09-01

**Authors:** Fang Wu, Wanjiang Zhang, Le Zhang, Jiangdong Wu, Chunzhu Li, Xianjie Meng, Xi Wang, Peng He, Jie Zhang

**Affiliations:** ^1^Department of Pathophysiology, Tongji Medical College, Huazhong University of Science and Technology, Wuhan 430030, China; ^2^Key Laboratory of Xinjiang Endemic and Ethnic Diseases Cooperated by Education Ministry with Xinjiang Province, Shihezi University, Shihezi 832002, China

## Abstract

*Background*. To explore the potential role of natural-resistance-associated macrophage protein 1 (NRAMP1) gene, vitamin D receptor (VDR) gene, (human leukocyte antigen, (HLA-DRB1) HLA) -DRB1 gene, and HLA-DQB1 gene polymorphisms in susceptibility to tuberculosis (TB) in the Chinese Kazakh population. *Methods*. A case-control study was performed on the Chinese Kazak population. Genetic polymorphisms of NRAMP1 gene (3′UTR) and VDR gene (TaqI and FokI) were analysed using polymerase chain reaction-restriction fragment length polymorphism (PCR-RFLP) and sequencing analysis in TB patients and healthy controls. Genetic polymorphisms of HLA-DRB1 gene and HLA-DQB1 gene in the two groups were detected with polymerase chain reaction-sequence-specific primers (PCR-SSPs) technique and sequencing analysis. *Results*. There was statistically significant difference in the 3′UTR polymorphism between the TB patients and healthy controls in the Chinese Kazak population (*P* = 0.002; OR = 1.859; 95% CI = 1.182–2.926). Significant difference was observed in the FokI polymorphism between the TB patients and healthy controls (*P* = 0.001; OR = 1.530; 95% CI = 1.007–2.325). It does not disclose any significant association between the disease and TaqI (*P* > 0.05). Alleles HLA-DRB1∗04 and HLA-DQB1∗0201 occurred more frequently in patients than in controls (*P* = 0.011 and 0.002; OR = 1.889 and 1.802; 95% CI = 1.153–3.095 and 1.230–2.639, resp.). *Conclusions*. Polymorphisms in the NRAMP1 gene, VDR gene, HLA-DRB1 gene, and HLA-DQB1 gene are statistically associated with susceptibility to TB in the Chinese Kazakh population.

## 1. Introduction

Tuberculosis (TB) remains a global health problem with 9.4 million new cases and 1.7 million deaths from TB a year. It is estimated that 1/3 of the world's population has been infected with *Mycobacterium tuberculosis*, and only a small proportion (10%) of those infected will develop to clinical disease during their life time. In addition, more than half of all TB cases occurred in Asia in 2009, which is far higher than other areas [1].

And ninety-five percent of TB cases and 98% of TB deaths are in developing countries [2]. Meaningfully, the incidence of TB is different in different countries, ethnic groups and populations [3]. China ranks the second in the world in the incidence of TB. Especially, the incidence of TB in the Xinjiang Uygur Autonomous Region (Northwest China) is higher than the average level of China. The TB epidemic situation in minority is significantly higher than that of the Han. For example, the prevalence rate of active, sputum smear, and culture positive TB in minority of the Xinjiang Uygur Autonomous Region was 12.4, 16.9, and 18.4 times that of the Han, respectively [4].

Many lines of evidence support a significant role for host genetic variation in susceptibility to TB [5, 6], and a complex interaction of genetic and environmental factors cause the development of clinical TB. A number of genes are thought to be important in the pathogenesis of TB [7, 8].

There is substantial evidence from studies on racial variation in susceptibility to TB [9, 10]. Polymorphisms in some genes have been found to be associated with TB in different ethnic groups. However, the results have been inconclusive. The specific aim of this study was to investigate the associations of NRAMP1 gene, VDR gene, HLA-DRB1 gene, and HLA-DQB1 gene polymorphisms in susceptibility to TB in the Chinese Kazak population. The difference between frequencies of these gene polymorphisms discovered susceptibility to TB and supplies the experimental data for TB risk assessment in the Chinese Kazak population.

## 2. Methods

### 2.1. Cases and Controls

Kazakh Chinese populations without miscegenation were selected from Tacheng region in the Xinjiang Uygur Autonomous Region of China, TB patients from the Tacheng District Hospital. All subjects agreed to take part in the study. Participants' eligibility was confirmed by questionnaire and clinical examinations after enrollment in the study. The cases were selected according to the national diagnostic criteria of China, with positive sputum smear or culture, or significant symptoms of typical PTB, chest radiography consistent with active disease, and a positive tuberculin skin test in case of negative sputum smear or culture. A control group composed of ethnically matched, unrelated individuals were also included in the analyses. None of the controls showed any clinical manifestations of PTB at the time of blood sample collection, and all were confirmed to be free from PTB by X-ray examination. Subjects, who were HIV positive and known to present any autoimmune, chronic inflammatory, or any other disease conditions, were excluded from the study. All selected participants had no mixed descendants within three generations. The study was approved by the Ethics Committee of the Faculty of Medicine (Huazhong University of Science and Technology, China), and informed consents were obtained from all subjects before blood sampling. Venous blood (3.0 mL) with anticoagulant was preserved in −80°C freezer for DNA extraction and detection of gene polymorphism.  Table 1 shows how many of the samples from recruited cases and controls could be utilized in the NRAMP1, VDR, and HLA genotyping experiments, respectively. 

The patients aged from 18 to 72 years. We used the comparison design and analysis. The result shows that there is no age and sex statistical difference between TB patients and healthy controls.

### 2.2. Genotyping of NRAMP1 and VDR Gene

Genomic DNA was extracted using the phenol-chloroform method [11].

The present study was carried out to determine the distribution of gene polymorphisms using a PCR-based restriction analysis. For the NRAMP1 gene, 3′UTR polymorphism site was investigated. For the VDR gene, TaqI and FokI polymorphism sites were detected.

Polymerase chain reaction (PCR) and restriction fragment length polymorphism (RFLP) analysis were used to type polymorphisms of NRAMP1 and VDR genes. The primers used for amplification of NRAMP1 and VDR gene polymorphisms were designed using the prime 5.0 (Table 2). PCR amplifications were performed using purified DNA in 25 *μ*L reaction volumes. Thermocycling parameters were as follows: 95°C for 5 min and 35 cycles of 95°C for 30 s, 58°C for 45 s, and 72°C for 45 s, with a final extension at 72°C for 10 min. PCR products were then digested using 1.0 *μ*L (10 U/*μ*L) of restriction endonuclease per reaction under the conditions recommended by the manufacturer (Takara, Kyoto, Japan). Digestion products were separated by electrophoresis on 2.5% agarose gel, stained with ethidium bromide, and visualised under ultraviolet light. The genotypes were defined according to generated fragment patterns. The restriction endonuclease used in each polymorphism and the defined genotypes are listed in Table 2. 10% PCR products were verified by DNA sequencing (Shanghai GeneCore Bio Technologies Co. Ltd.).

### 2.3. Genotyping of HLA-DRB1 and HLA-DQB1

All samples were typed for HLA-DRB1 and HLA-DQB1 using PCR-SSP, The primer sequence and product size are listed in Table 3.

A conserved sequence of human growth hormone (HGH) was amplified in each reaction as an internal control primer; former and reverse primer sequences are 5-GCCTTCCCAACCATTCCCTTA-3 and 5-TCACGGATTTCTGTTGTGTTTC-3. PCR amplifications were performed in 25 *μ*L reaction volumes containing 100 ng genomic DNA, 50 mM Tris-HCl, 2.5 mM MgCl_2_, 20 mM each deoxynucleotide triphosphate, 10 pmol each primer, and 1 U of Taq DNA polymerase. Thermocycling parameters were as follows: denaturation at 94°C for 5 min and 30 cycles of 94°C for 30 s, 60°C for 45 s, and 72°C for 45 s, with a final extension at 72°C for 5 min. Amplified products were resolved using agarose (Roche, Mannheim, Germany) gel electrophoresis. The genotypes were defined according to generated fragment patterns. 10% PCR products were verified by DNA sequencing (Shanghai GeneCore Bio Technologies Co., Ltd.).

### 2.4. Quality Control

All tests of genetic polymorphisms were processed by sophisticated laboratory personnel. The samples were reexamined if results were inconsistent.

### 2.5. Data and Statistical Analysis

Data were managed and analysed using SPSS (version 13.0). For each polymorphism, allele and genotype frequency differences in each group were examined by the chi-squared test or Fisher's exact test when appropriate. Odds ratios (ORs) and 95% confidence intervals (CIs) were calculated to quantitatively assess the degree of association between these polymorphisms and tuberculosis. Hardy-Weinberg equilibrium (HWE) was tested for each SNP. Differences were considered statistical significance at *P* < 0.05.

## 3. Results

### 3.1. Distribution of the Selected NRAMP1 and VDR Gene Polymorphisms

The distribution of NRAMP1 and VDR polymorphisms in 424 Chinese Kazakhs did not deviate from the Hardy-Weinberg equilibrium (*P* > 0.05). The frequencies of 3′UTR, FokI, and TaqI SNP in patients with TB were 35.2%, 66.2%, and 10.3%, respectively, whereas the frequencies in healthy controls were 20.9%, 52.1%, and 13.3%, respectively (Table 4). 

The TGTGdel, f, and t allele frequencies of 3′UTR, FokI, and TaqI SNP in patients with TB were 20.4%, 43.7%, and 5.9%, respectively, whereas the allele frequencies in healthy controls were 11.1%, 31.3%, and 7.8%, respectively (Table 4).

The data showed that in Kazak population, the TGTGdel and f allele frequencies of the 3′UTR and the FokI SNPs in the patient groups were higher than in the control groups, indicating significant differences between the two groups (*P* = 0.000 and 0.000, resp.). No significant difference in the t allele frequency was observed between the TB patients and the controls (*P* = 0.260) (Table 4). 

Our results showed that the polymorphisms of the NRAMP1 gene (3′UTR) and VDR gene (FokI) are significantly associated with TB and may be risk factors for the development of TB in the Chinese Kazak population (OR = 1.859 and 1.530; *P* = 0.002 and 0.001, resp.). Our results indicated that the polymorphism in the TaqI of the VDR gene is not associated with TB (*P* = 0.581).

### 3.2. Distribution of the Selected HLA-DRB1 and HLA-DQB1 Gene Polymorphisms

The distribution of HLA-DRB1 and HLA-DQB1 polymorphisms in 461 Chinese Kazakhs did not deviate from the Hardy-Weinberg equilibrium (*P* > 0.05). The frequencies of HLA-DRB1*04 genotype were 6.75% in control group, and 11.72% in TB group. HLA-DRB1*04 genotype had a significant increased risk of TB (OR = 1.889; *P* = 0.011). The frequency of HLA-DQB1*0201 genotype was 16.9% in control group and 25.6% in TB group. HLA-DRB1*0201 genotype had a significant increased risk of TB (OR = 1.802; *P* = 0.002) (Table 5). 

## 4. Discussion

The association of host genetic factors with susceptibility or resistance to TB has been studied extensively using various methods, such as case-control studies, candidate gene approaches and family-based, and genome-wide linkage analyses that have revealed several important candidate genes for susceptibility [12–14].

Solute carrier family 11A member 1 (SLC11A1), formerly known as natural resistance-associated macrophage protein 1 (NRAMP1), is a human homologue of the mouse Nramp1 gene, and a single nonconservative amino acid substitution in this protein controls susceptibility to *Leishmania*, *Salmonella*, and *Mycobacteria* in inbred mouse strains [15]. SLC11A1 activates microbicidal responses in the infected macrophage, and it is, therefore, important in the early innate response to mycobacterial infection. Its exact function is still unclear, but the fact that it is known to find in the late endosome fraction [16], and some of its homologues have been shown to be divalent cation transporters, and has led to speculation that Nramp1 may control intracellular microbial replication by actively removing iron or other divalent cations from the phagosomal space [17, 18]. While iron is an essential mycobacterial nutrient, it is also required by the cell to generate reactive oxygen and nitrogen intermediates. Divalent cations are also essential cofactors for enzymes, such as superoxide dismutase and catalase, which neutralize the cytotoxic effects of the oxidative burst in macrophages [19]. NRAMP1 polymorphic variants have been associated with susceptibility to TB and leprosy as well as autoimmune diseases [20, 21].

3′UTR has a 4 bp TGTG deletion located 55 nt downstream of the last codon in exon 15. In the Chinese Kazak population, the frequency of 3′UTR TGTG+/del genotype was significantly higher in the patient group than in the control group, indicating that the TGTG+/del genotype frequency is associated with an increased risk of TB. There was a significant difference between TB patients and healthy controls. Our date also showed that the frequency of 3′UTR TGTGdel/del genotype was three times as high in the patient group than in the control group, indicating that the TGTGdel/del genotype frequency is associated with an increased risk of TB. There was significant difference between TB patients and healthy controls.

Four NRAMP1 polymorphisms (3′UTR, INT4, D543N, and 5′(GT)n) were found to be significantly associated with tuberculosis in West Africans [22]. In Koreans [23, 24] and Chinese Han [25], a significant association with TB was detected for 3′UTR polymorphism. However, in Cambodians, the 3′UTR variants were associated with resistance to TB [26]. In Taiwanese [27], Thai [28], Moroccans [29], Danes [30], and Brazilian [31], there were no associations between the 3′UTR variants and TB. In the present study, we found that the 3′UTR polymorphisms have a relatively high risk for TB in the Chinese Kazak population (OR = 1.859; 95% CI = 1.182–2.926; *P* = 0.002). The results presented here show that in the Chinese Kazak population there were associations of the 3′UTR variants with susceptibility to TB.

Vitamin D metabolism can lead to the activation of macrophages and subsequently restrict the intracellular growth of M. TB [32, 33]. This effect is achieved by binding to VDR in the monocytes, and the polymorphism in the VDR gene is suggested to be involved in genetic susceptibility to TB.

In the Chinese Kazak population, the frequencies of FokI-Ff and ff genotypes were higher in the patient group than in the control group, indicating that the Ff and ff genotype frequencies are associated with an increased risk of TB. There was a significant difference between TB patients and healthy controls. No significant differences of TaqI-Tt and tt genotype frequencies were observed between TB patients and healthy controls.

The relation between VDR gene polymorphism and susceptibility to TB has been studied in different populations. Polymorphisms of the VDR gene have been associated with TB resistance in The Gambia, and data showed a significantly lower frequency of TaqI-tt genotype among TB patients [34]. Among Gujarati Asians in West London [35], a significant interaction between vitamin D status and FokI and TaqI genotypes was also observed. In Tuvinians from Tuva Republic and Russians from Tomsk City, no association between FokI genotype and PTB was found [36]. A study in Cambodia [26] and Tanzania [37] found no association between VDR gene and PTB. In Chinese Han population [25], FokI-ff is associated with susceptibility to PTB, but there is no any significant association between the disease and TaqI. In this study, we found that the FokI polymorphisms have a relatively high risk for TB in the Chinese Kazak population (OR = 1.530; 95% CI = 1.007–2.325; *P* = 0.001). The FokI polymorphisms were associated with TB in the Chinese Kazak population, but there is no any significant association between the disease and TaqI. 

The aim of the present study is to report that the polymorphisms of the NRAMP1 gene (3′UTR) and VDR gene (FokI) are significantly associated with TB and may be a risk factor for the development of TB in the Chinese Kazak population. But TaqI is not associated with the TB patients and healthy controls.

The human leucocyte antigen (HLA) system plays an important role in the modulation of the immune response. Epidemiological and experimental studies suggest that the high degree of molecular diversity in HLA molecules influences the variability in the human response to TB. An association between HLA and TB has been examined in several populations, but the results have been inconsistent. The HLA class II variant, DR2 encoded by alleles DRB1*15 and DRB1*16, is associated with TB in several populations [38, 39], but not in others [40, 41]. In South Africans [42], a significant interaction between HLA-DRB1*1302 allele and susceptibility to TB was observed. A study in Poland [43, 44] showed that there was an interaction between HLA-DQB1*05 allele and susceptibility to TB and a significant interaction between HLA-DRB1*16 and HLA-DRB1*14 allele and susceptibility to TB was also observed. In Koreans [45], a significant interaction between HLA-DQB1*0601 allele and susceptibility to TB was observed. In Mexcians [46], a significant interaction between HLA-DQ genotype and susceptibility to TB was also observed. The less common DQB1*0503 HLA class II allele associated with TB in Cambodia [41] was shown. In this study, the HLA-DRB1*04 and HLA-DQB1*0201 alleles were associated with TB (OR = 1.889 and 1.802; 95% CI = 1.153–3.095 and 1.230–2.639; *P* = 0.011 and 0.002, resp.); the results were different from other ethnic groups.

Gene polymorphisms are influenced by different ethnic, geographical, and other factors. Differences in genetic background among different races have also led to the genotype frequency distribution that appeared quite different. Sample size, case and control groups of sources, inclusion criteria, and different technical factors may also lead to different results. Further studies are required on the function of these genes. Further analyses are necessary to clearly elucidate the mechanisms by which the gene might affect disease progression. The findings of this study provide data for the assessment of risk profiles regarding susceptibility to TB. We anticipate that the determination of ethnic-specific genetic associations with TB susceptibility may guide TB therapy and prophylaxis in an ethnic-specific manner.

## Figures and Tables

**Table 1:** TB cases and controls included in the analyses of NRAMP1, VDR, and HLA as candidate genes in TB susceptibility.

	NRAMP1 genotyping	VDR genotyping		HLA genotyping	
	3′UTR	FokI	TaqI	DRB1	DQB1
TB cases	213	213		231	
Controls	211	211		230	

**Table 2:** NRAMP1 and VDR polymorphism sites and sequence variants.

Name	Nucleotide and amino acid change	Primers, 5′ to 3′	Polymorphic site	Genopytes	Fragment (bp)
NRAMP1- 3′UTR	Deletion of TGTG in the 3′UTR (55 nt 3′ to the last codon in exon 15)	GCATCTCCCCAATTCATGGT		TGTGdel/del	240
		AACTGTCCCACTCTATCCTG	TGGA **T** GTGGA	TGTG+/del	240, 211, 33
		(240 or 242 bp)	FokI	TGTG+/+	211, 33
VDR-FokI	A T/C transition polymorphism in exon 2	AGCTGGCCCTGGCACTGACTCTGCTCT		C/C-FF	267
		ATGGAAACACCTTGCTTCTTCTCCCTC	CAGGGA **T** GGAGG	T/C-Ff	267, 197, 70
		(267 bp)	FokI	T/T-ff	197, 70
VDR-TaqI	A single base change	CAGAGCATGGACAGGGAGC		T/T-TT	455
	C to T in codon 352 at the 3′ end of the	AGGAGAGGCAGCGGTACTG	ACAGGAG **C** TCT	T/C-Tt	455, 290, 165
	VDR gene	(455 bp)	TaqI	C/C-tt	290, 165

Note: Underlined letters denote different restriction sites in which bold letters represent polymorphism sites. Genotypes of both genes were defined as follows. For the NRAMP1 gene, individuals were scored as 3′UTR-TGTG+/+ wild homozygote, 3′UTR-TGTGdel/del mutant homozygote, and 3′UTRTGTG+/del heterozygote, respectively.

+= presence of TGTG; del = absence of these four bases.

For the VDR gene, individuals were scored as FokI-FF, TaqI-TT wild homozygotes, FokI-ff, TaqI-tt mutant homozygotes, FokI-Ff, and TaqI-Tt heterozygotes, respectively.

**Table 3:** The primers and product size for PCR-SSP genotyping of HLA-DRB1 and HLA-DQB1.

	Primer sequence (5′ to 3′)		Size of PCR products (bp)
	For	Rev	
DRB1 allele			
01	TTGTGGCAGCTTAAGTTTGAAT	CTGCACTGTGAAGCTCTCAC	255
03	TACTTCCATAACCAGGAGGAGA	TGCAGTAGTTGTCCACCCG	151
04	GTTTCTTGGAGCAGGTTAAACA′	CTGCACTGTGAAGCTCTCAC	260
07	CCTGTGGCAGGGTAAGTATA	CCCGTAGTTGTGTCTGCACAC	232
08	AGTACTCTACGGGTGAGTGTT′	CTGCAGTAGGTGTCCACCAG	214
09	GTTTCTTGAAGCAGGATAAGTTT	CCCGTAGTTGTGTCTGCACAC	236
10	CGGTTGCTGGAAAGACGCC	CTGCACTGTGAAGCTCTCAC	204
11	GTTTCTTGGAGTACTCTACGTC	CTGGCTGTTCCAGTACTCCT	176
12	AGTACTCTACGGGTGAGTGTT	CACTGTGAAGCTCTCCACAG	248
13	ACTTCCATAACCAGGAGGAGA	CCCGCTCGTCTTCCAGGAT	130
14	GTTTCTTGGAGTACTCTACGTC	TCTGCAATAGGTGTCCACCT	224
15	TCCTGTGGCAGCCTAAGAG	CCGCGCCTGCTCCAGGAT	197
16	TCCTGTGGCAGCCTAAGAG	AGGTGTCCACCGCGGCG	223
DQB1 allele			
0201	GTGCGTCTTGTGAGCAGAAG	GCAAGGTCGTGCGGAGCT	205
0301/4	GACGGAGCGCGTGCGTTA	AGTACTCGGCGTCAGGCG	122
0302/3	GACGGAGCGCGTGCGTCT′	AGTACTCGGCGTCAGGCG	122
0303	GACGGAGCGCGTGCGTCT	CTGTTCCAGTACTCGGCGT′	129
0304	GACGGAGCGCGTGCGTTA	CTGTTCCAGTACTCGGCGG	129
0401	CACCAACGGGACCGAGCT	GGTAGTTGTGTCTGCATACG	200
0402	CACCAACGGGACCGAGCG	GGTAGTTGTGTCTGCATACG	200
0501	CGGAGCGCGTGCGGG	GCTGTTCCAGTACTCGGCAA	128
0502	TGCGGGGTGTGACCAGAC	TGTTCCAGTACTCGGCGCT	117
0503	TGCGGGGTGTGACCAGAC	GCGGCGTCACCGCCCGA	87
0504	GTGCGGGGTCTGACCAGAT	TGTTCCAGTACTCGGCGCT	118
0601	GCCATGTGCTACTTCACCAAT	CACCGTGTCCAACTCCGCT	198
0602	CGTGCGTCTTGTGACCAGAT	GCTGTTCCAGTACTCGGCAT	121
0603/8	GGAGCGAGTGCGTCTTGTA	GCTGTTCCAGTACTCGGCAT	127
0604	CGTGTACCAGTTTAAGGGCA	GCAGGATCCCGCGGTACC	254

Note: For: former; Rev: reverse.

**Table 4:** Genotype and allele frequency distribution of NRAMP1 and VDR genes in the controls and TB patients of the Chinese Kazak population.

Genotype and allele	Tuberculosis *N* (Freq)	Control *N* (Freq)	*χ* ^2^	OR (95% CI)	*P*
NRAMP1-3′UTR					
TGTG++	138 (64.8)	167 (79.1)		1.00	
TGTG+/del	63 (29.6)	41 (19.5)	12.802	1.859 (1.182–2.926)	0.002
TGTGdel/del	12 (5.6)	3 (1.4)		4.841 (1.339–17.499)	
Alleles					
TGTG	339 (79.6)	375 (88.9)	13.737	2.048 (1.395–3.006)	<0.001
TGTGdel	87 (20.4)	47 (11.1)			
VDR-FokI					
FF	72 (33.8)	101 (47.9)		1.00	
Ff	96 (45.1)	88 (41.7)	13.095	1.530 (1.007–2.325)	0.001
ff	45 (21.1)	22 (10.4)		2.869 (1.586–5.191)	
Alleles					
F	240 (56.3)	290 (68.7)	13.868	1.703 (1.285–2.255)	<0.001
f	186 (43.7)	132 (31.3)			
VDR-TaqI					
TT	191 (89.7)	183 (86.7)		1.00	
Tt	19 (8.9)	23 (10.9)	—	0.791 (0.417–1.502)	0.581
tt	3 (1.4)	5 (2.4)		0.575 (0.135–2.440)	
Alleles					
T	401 (94.1)	389 (92.2)	1.267	0.735 (0.429–1.259)	0.260
t	25 (5.9)	33 (7.8)			

Note: Genotype frequencies were compared between the patients and control subjects by *χ* ^2^ test or Fisher's exact test when appropriate. Allele frequencies were compared between the patients and control subjects by use of a *χ* ^2^ test for 2-by-2 contingency tables. *N*: the sample size; OR: odds ratio; 95% CI: 95% confidence interval; Freq: frequency.

**Table 5:** The allele frequency distribution of HLA-DRB1 and HLA-DQB1 genes in the controls and TB patients of the Chinese Kazakh population.

Alleles	Tuberculosis N (Freq)	Control *N* (Freq)	*χ* ^2^	OR (95% CI)	P
HLA-DRB1					
01	19 (4.2)	17 (3.77)	0.111	1.123 (0.568–2.220)	0.739
03	12 (5.19)	7 (1.53)	1.350	1.746 (0.675–4.516)	0.245
04	51 (11.72)	30 (6.75)	6.495	1.889 (1.153–3.095)	0.011
07	30 (12.99)	24 (5.36)	0.726	1.281 (0.724–2.267)	0.394
08	17 (3.75)	14 (3.09)	0.297	1.226 (0.589–2.549)	0.585
09	7 (1.53)	12 (2.64)	1.395	0.568 (0.219–1.469)	0.238
10	13 (2.86)	5 (1.09)	3.664	2.683 (0.941–7.654)	0.056
11	3 (0.66)	8 (1.76)	2.351	0.365 (0.096–1.394)	0.125
12	19 (4.2)	13 (2.87)	1.181	1.496 (0.721–3.106)	0.277
13	15 (3.3)	12 (2.64)	0.340	1.262 (0.577–2.758)	0.560
14	16 (3.53)	10 (2.2)	1.440	1.637 (0.727–3.688)	0.230
15	42 (18.18)	31 (6.98)	1.913	1.427 (0.861–2.364)	0.617
16	8 (1.75)	6 (1.31)	0.286	1.339 (0.457–1.024)	0.593
HLA-DQB1					
0201	103 (25.6)	71 (16.9)	9.231	1.802 (1.230–2.639)	0.002
0301/4	16 (3.5)	13 (2.87)	0.317	1.242 (0.583–2.645)	0.573
0302/3	13 (2.9)	7 (1.53)	1.885	1.900 (0.744–4.852)	0.173
0303	22 (4.88)	18 (3.99)	0.419	1.240 (0.646–2.379)	0.517
0304	19 (4.2)	14 (3.09)	0.793	1.383 (0.676–2.829)	0.373
0401	24 (5.34)	25 (5.59)	0.028	0.951 (0.526–1.719)	0.867
0402	26 (5.79)	32 (7.21)	0.740	0.785 (0.451–1.364)	0.390
0501	47 (10.75)	41 (9.1)	0.474	1.177 (0.739–1.875)	0.491
0502	39 (8.83)	53 (12.27)	2.738	0.678 (0.428–1.076)	0.120
0503	11 (2.41)	9 (1.97)	0.200	1.228 (0.499–3.021)	0.655
0504	17 (3.75)	20 (4.45)	0.279	0.834 (0.425–1.637)	0.597
0601	51 (12.14)	36 (8.16)	3.108	1.527 (0.952–2.449)	0.078
0602	29 (6.49)	38 (8.63)	1.461	0.725 (0.430–1.223)	0.227
0603/8	14 (3.08)	23 (5.13)	2.423	0.581 (0.291–1.159)	0.120
0604	31 (6.95)	25 (5.59)	0.702	1.271 (0.725–2.229)	0.402

Note: *N*: the sample size; OR: odds ratio; 95%, CI: 95% confidence interval; Freq: frequency.
